# Diabetes and Chronic Pancreatitis: Considerations in the Holistic Management of an Often Neglected Disease

**DOI:** 10.1155/2019/2487804

**Published:** 2019-10-07

**Authors:** Philip C. Johnston, Judith Thompson, Allison Mckee, Connor Hamill, Ian Wallace

**Affiliations:** ^1^Diabetes Department, 51 Lisburn Road, BT9 7AB Belfast City Hospital, UK; ^2^Dietetics Department, 51 Lisburn Road, BT9 7AB Belfast City Hospital, UK

## Abstract

Diabetes secondary to chronic pancreatitis (CP) or type 3cDM refers to a brittle form of diabetes and is often characterised by hypoglycaemic episodes, erratic glycaemic control, and impaired quality of life. It differs from other forms of diabetes and is typically characterised by concurrent pancreatic endocrine and exocrine insufficiency which can present as malabsorption and nutritional deficiencies. In this review, we discuss the pathogenesis, epidemiology, and the practicalities of diagnosis, screening, and management of this condition.

## 1. Clinical Case

Mr. G is a 46-year-old male who was diagnosed with chronic pancreatitis (CP) 6 years prior to his outpatient clinic consultation. He had developed diabetes mellitus a year after his CP was diagnosed. He has a history of partial pancreatectomy due to pancreatic pseudocyst formation 4 years prior. The aetiology of his CP was from alcohol dependence. His last episodes of pancreatitis was in the previous year. His HbA1c is 75 mmol/mol, and he is maintained on a basal bolus regimen of Lantus and NovoRapid. His ACR and lipids are at target. His recent eye and feet screening testing is normal. He takes 50,000 units of Creon with his meals. He smokes 20 cigarettes per day and has been referred for smoking cessation. He has stopped alcohol one month prior to his clinic visit. He continues to experience intermittent abdominal pain and loose stools. He has had a weight loss of one stone over the previous 3 months due to inadequate glycaemic control and intermittent compliance with his Creon (PERT—pancreatic enzyme replacement therapy). After discussion with the patient regarding the benefits of compliance of his medications and the rationale for the importance of good glycaemic control, he reported a steady improvement in his quality of life. At a follow-up 6 months later and with improved compliance with PERT and engagement with dietary changes and titration of his insulin regimen, he has gained 5 kg with improvement in his gastrointestinal symptoms; glycaemic control has improved to a HbA1c of 67 mmol/mol.

## 2. Introduction

Pancreatogenic diabetes or type 3c diabetes mellitus (T3cDM) refers to diabetes arising from pancreatic disease and is characterised by pancreatic exocrine insufficiency (PEI). The prevalence of T3cDM is around 5-10% of diabetic populations with the majority (80%) arising from chronic pancreatitis (CP) [1]. Other common aetiologies of pancreatogenic diabetes include cystic fibrosis, haemochromatosis, pancreatic cancer, and pancreatectomy [2]. Common aetiologies of chronic pancreatitis include alcohol, gallstones, genetic (PRSS1, SPINK1, and CFTR mutations), and autoimmune [3]. The pancreas in chronic pancreatitis is characterised by an inflammatory response with infiltration of inflammatory cells and release of proinflammatory cytokines and activation of pancreatic stellate cells with synthesis of extracellular matrix proteins and fibrogenesis; the resulting fibrosis leads to progressive and eventual exocrine and endocrine cell destruction [4]. Exocrine insufficiency typically presents earlier in the disease process and can manifest as abdominal pain, malabsorption, nutritional deficiencies, and sarcopenia. T3cDM in chronic pancreatitis is a result of complex endocrine physiology, mainly as a consequence of insulin deficiency from acinar cell fibrosis resulting in reduced production of insulin with resultant hyperglycaemia. Primarily, the islet cell loss affects the beta cells. Other cell types in CP can be affected, including alpha and delta cells as well as PP (pancreatic polypeptide) cells which give CP-DM a unique pathophysiology [5, 6]. As the disease progresses, the diabetes in CP-DM tends to be brittle with impaired glucagon secretion and susceptibility to hypoglycaemia. Diabetes may be the first clinical manifestation of chronic pancreatitis; conversely, it can also be a late complication of chronic pancreatitis.

## 3. Diagnosis, Imaging, and Screening

Distinguishing T3cDM from other aetiologies of diabetes can be challenging. There is no standardised diagnostic criteria for T3cDM secondary to CP. Type 3c diabetes is frequently misclassified as type 2 diabetes. Ewald and Hardt have outlined three major criteria for the diagnosis of T3cDM, all of which must be fulfilled: (1) the presence of pancreatic exocrine insufficiency, (2) pathological pancreatic imaging, and (3) the absence of T1DM autoantibodies (Table 1). Minor criteria include impaired *B* cell function, low levels of fat-soluble vitamins (A, D, E, and K), lack of excessive insulin resistance, and impaired incretin release or pancreatic polypeptide secretion [7]. Typically, the pancreatic polypeptide (PP) response is blunted during mixed meal testing in T3cDM [8].

**Figure 1:**
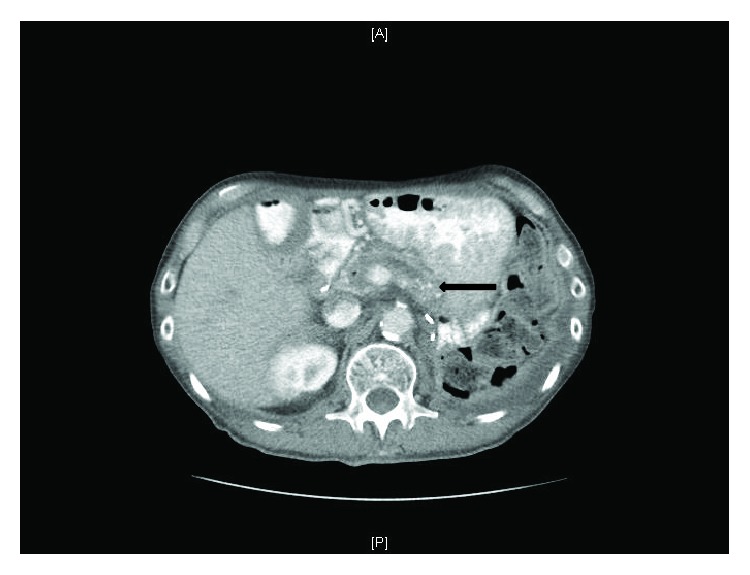
CT imaging. Evidence of severe chronic pancreatitis with calcifications (arrow) and dilation of the pancreatic duct.

Morphological evaluation of the pancreas in CP includes abdominal ultrasound and computed tomography (CT), which can be accurate for detecting calcifications (Figure 1) and main pancreatic duct dilation but has low sensitivity for mild to moderate CP changes [9]. MRI and MRCP (and dynamic MRCP with secretin) are typically utilized for the diagnosis of advanced calcified CP, parenchymal atrophy, pseudocysts, and dilation and irregularity of the main pancreatic duct and side branches [10]. In addition to the use as an indirect method to predict PEI, endoscopic ultrasound (EUS) is useful in detecting minimal change or noncalcified CP as well as for detecting and biopsy of pancreatic masses [11]. ERCP is also quite sensitive for CP in addition to its use for interventional options [12].

Risk factors for the development of diabetes in patients with chronic pancreatitis include smoking, longer duration of disease, previous pancreatic surgery, and the presence of calcifications on pancreatic imaging [13–15]. Screening for diabetes (or prediabetes) can be performed annually in patients with chronic pancreatitis and can include fasting and/or random glucose, HbA1c, and OGTT. The diagnostic criteria for the diagnosis of diabetes in patients with chronic pancreatitis are the same as that of T1DM and T2DM; the American Diabetes Association guidelines give values of a fasting plasma glucose of >7 mmol/l or higher, a 2-hour plasma glucose level of 11.1 mmol/l or higher during a 75 g oral glucose tolerance test, and a random glucose of 11.1 mmol/l or higher as well as a HbA1c of >48 mmol/mol for a diagnosis of diabetes [16]. Functional *B* cell mass can be measured from serum C-peptide levels during oral glucose tolerance or mixed meal tolerance tests [17, 18].

## 4. Endocrine Complications

There is limited data on the rates of hypoglycaemia and ketoacidosis in CP-DM. Hypoglycaemia episodes in CP-DM can be common and are often prolonged due to impaired glucagon secretion. The aetiology of hypoglycaemia in CP-DM is multifactorial and includes the use of insulin, impaired counterregulatory responses, glycogen storage deficits, malnutrition, and malabsorption as well as alcohol consumption [19]. Although long-term data is limited and consensus guidelines on CP-DM patients are lacking, there appears to be a similar risk for micro- and macrovascular complications in comparison to T1DM and T2DM. In the large clinical trials (mainly DCCT and UKPDS), patients with CP-DM were generally excluded. In the only prospective study to date of 54 patients with CP-DM, the risk of diabetic retinopathy was 31% [20]. In other separate studies, the rate of diabetic retinopathy was 37%, nephropathy was 29%, and peripheral vascular disease was 26% [21–23]. Chronic pancreatitis is associated with a poor prognosis, with increased morbidity and mortality including an increased risk of pancreatic cancer, especially alcoholic pancreatitis, and therefore, long-term complications including retinopathy and nephropathy in some patients do not develop because of reduced life expectancy. In common with T1DM and T2DM, cardiovascular risk factors and lifestyle modifications should be addressed including smoking and alcohol consumption, obesity, hypertension, and hyperlipidaemia. Another important consideration is the development of metabolic bone disease and osteoporosis [24, 25]; vitamin D testing and supplementation if deficient should be considered in addition to its possible benefit on impaired incretin release in CP-DM [26]. Patients should be monitored and followed up in the same way as T1DM and T2DM for the development of nephropathy, retinopathy, and neuropathy.

## 5. Pancreatic Exocrine Insufficiency

Pancreatic exocrine insufficiency (PEI) is characterised by a deficiency of exocrine pancreatic enzymes which results in malabsorption, malnourishment, and nutrient deficiencies of fat-soluble vitamins. PEI usually precedes DM in chronic pancreatitis but can often be underdiagnosed and undertreated [27]. Malabsorption symptoms include abdominal pain, flatulence, weight loss, and steatorrhea. Malabsorption can be masked by medications, low-fat diets, and poor oral intake. It is estimated that around 80-90% of patients with CP will have some degree of PEI [28]. Testing for PEI includes the gold standard of the 72-hour faecal fat test which can be time-consuming and impractical for patients, the faecal elastase-1 test which is not as sensitive in mild to moderate PEI (200-500 *μ*g/g), and the ^13^C-mixed triglyceride (^13^C-MTG) breath test, which is only available in limited centres [29–31]. PERT (pancreatic enzyme replacement therapy) replaces the digestive enzymes that are lost to PEI. PERT is taken in a capsule from typically Creon. The usual starting dose is 40-50,000 units with meals; a total of 10-25,000 is taken for snacks [32]. The dose is adjusted until the symptoms of PEI resolve. A referral to a dietitian is essential and is the cornerstone of management in patients with CP. Fat-soluble vitamins may be supplemented if required. PERT has been shown to improve glycaemic control and also to reduce episodes of mild to moderate hypoglycaemia [33].

## 6. Pancreatic Cancer

Around 80% of pancreatic cancer patients have glucose intolerance or overt diabetes. This association has led to two hypotheses: (1) that pancreatic cancer causes diabetes and (2) that diabetes is a risk factor for the development of pancreatic cancer [34]. Numerous studies have been conducted examining these relationships, with evidence supporting both of these hypotheses; however, the relationship between glucose metabolism and pancreatic cancer remains complex. Chronic pancreatitis and diabetes are both associated with an increased risk of pancreatic cancer, and the degree of hyperglycaemia appears to be related to the risk of developing cancer [35, 36]. There are suggestions that insulin therapy, sulphonylurea therapy, and sitagliptin or exenatide can confer an increased risk of pancreatic cancer. Bonelli et al. found that diabetes was associated with a 2.86-fold increase in the risk of pancreatic cancer, the risk increasing to 6.49-fold for those treated with insulin, compared to 2.12-fold for those treated with oral hypoglycaemic agents [37]. Pancreatic cancer is also a cause of diabetes due to unknown mechanisms. Differentiating new-onset diabetes as a result of pancreatic cancer may allow for earlier diagnosis and intervention in early-stage disease. Lee et al. have suggested that in distinguishing pancreatic cancer-associated diabetes (type 3cDM) and T2DM, a lack of family history, age 65 years or older, recent weight loss of >2 kg, and a BMI < 25 kg/m^2^ suggest that type 3c is more likely [38]. Metformin is used in patients with CP and has been associated with a reduced risk of pancreatic cancer due to its antitumour effects [39]. Studies on the use of biomarkers for screening for pancreatic cancer-associated diabetes are ongoing at present.

## 7. Management

### 7.1. General Considerations and PERT

Management of CP-DM is challenging and ideally should require multidisciplinary input including a diabetologist, specialist diabetes nurses, diabetes specialist dietitian (preferably with pancreatic experience), and gastrointestinal, hepatobiliary, and pain services [40]. The management of CP-DM can be difficult due to chronic pain, analgesia side effects, nausea, variable nutritional intake, and alcohol consumption. An individualised approach is required, and patients with CP-DM ideally should be best managed in dedicated clinics with clinicians who have an interest and expertise with this condition. Currently, there are no specific guidelines to manage T3cDM in chronic pancreatitis, although there have been recommendations from various consensus groups [41, 42]. Lifestyle modifications include the avoidance of toxic precipitants including smoking and alcohol as well as regular exercise. Specialist dietetic support should be provided with the goals of controlling symptoms of malabsorption and maintaining normoglycaemia. Patients should be encouraged to have a well-balanced diet with no unnecessary fat or carbohydrate restriction. If pain is a constant feature of the disease, referral to a suitable specialist pain clinic is desired. If PEI is present, PERT therapy should be instituted as well as replacement of fat-soluble vitamins if necessary. Screening for the identification of micro- and macrovascular complications should be performed routinely.

### 7.2. Hypoglycaemic Therapy

Treatment goals will vary between patients and will depend on their diagnosis, nutritional status, lifestyle, and comorbidities. Glycaemic targets include a fasting serum glucose of 3.9-7.2 mmol/l and HbA1c of 53 mmol/mol; if hypoglycaemic is frequent, glycaemic controls should be relaxed [43]. There are no current guidelines for the use of antihyperglycaemic agents in CP-DM; research into the optimum treatment strategies for type 3c is lacking. If hyperglycaemia is mild and if insulin resistance is present, metformin should be considered. In addition to its antihyperglycaemic effects, it also can reduce the risk of pancreatic cancer in this population [39]. Adverse side effects including weight loss and gastrointestinal upset are undesirable in CP, and patients should be counselled on the potential side effects. Sulphonylureas are associated with hypoglycaemia and are not used as first line therapy in CP-DM. Thiazolidinediones (TZD) are generally avoided due to increased risk of fractures, fluid retention, and heart failure and are not routinely used. Incretin-based therapies (GLP-1 analogs and DPP-IV inhibitors) enhance insulin secretion and have potential in beta cell mass preservation but reduce appetite and can lead to weight loss which is not desired in this population. The possible association with pancreatic cancer and acute pancreatitis would suggest that these medications should not be recommended. There is no data on the use of sodium-glucose cotransporter 2 inhibitors (SGLT2), with the risk of dehydration and weight loss possibly precluding their use [4].

As the main defect in CP-DM is insulin deficiency, for most patients, insulin is the mainstay of treatment and is initiated when oral therapy has not worked or there is uncontrolled hyperglycaemia. Insulin can increase the risk of malignancy in addition to the side effects of weight gain and hypoglycaemia. In advanced CP, basal bolus insulin regimen should be used; consideration should be given to carbohydrate awareness education, continuous glucose monitoring, and insulin pumps in selected patients.

### 7.3. Autologous Islet Cell Transplantation

Total pancreatectomy with autologous islet cell transplantation (TP-AIT) can relieve pain and preserve beta cell mass in patients with CP when other treatment modalities have failed. It is employed in some centres but with limited numbers and has been shown to improve quality of life and maintain reasonable glycaemic control in certain patients, with benefits seen when the procedure is performed earlier in the disease process, but can be associated with spontaneous hypoglycaemia [44–46].

## 8. Summary

As characterised by our representative patient presented in the clinical vignette, patients with CP-DM tend to have an impaired quality of life, with variable glycaemic control, nutritional deficiencies, and malabsorption. Therefore, the management of CP-DM is challenging and should encompass a multidisciplinary team to deal with short-term and to prevent long-term complications.

## Figures and Tables

**Table 1:** Proposed diagnostic criteria for type 3c diabetes mellitus.

Major criteria
(i) Presence of exocrine pancreatic insufficiency (faecal elastase)
(ii) Pathological pancreatic imaging (endoscopic ultrasound (EUS), MRI, and CT)
(iii) No type 1 diabetes mellitus-associated autoimmune markers
Minor criteria
(i) The absence of pancreatic polypeptide (PP) secretion
(ii) Impaired incretin secretion
(iii) No excessive insulin resistance (HOMA-IR)
(iv) Impaired beta cell function (HOMA-B, C-peptide/glucose-ratio)
(v) Low serum levels of lipid soluble vitamins (A, D, E, and K)
